# DECLINES IN CONTROL BELIEFS OVER TIME: THE ROLE OF PERCEIVED DISCRIMINATION AND INEQUALITY

**DOI:** 10.1093/geroni/igad104.3077

**Published:** 2023-12-21

**Authors:** Stefan Agrigoroaei, Denisa Cristina-Alina Berceanu

**Affiliations:** Universite Catholique de Louvain, Louvain-la-Neuve, Brabant Wallon, Belgium; University of Bucharest, Bucharest, Bucuresti, Romania

## Abstract

Higher control beliefs represent a key protective factor for healthy aging, broadly defined. If the benefits of control beliefs are well documented, fewer studies have focused on the antecedents of changes in control. There is empirical evidence that social stress, such as being treated unfairly or discriminated against, may erode personal control. The goal of this study was to examine perceived discrimination and inequality in relation to long-term change in control beliefs, in a national sample, using data from the Midlife in the United States (MIDUS) longitudinal study (Waves II and III, N = 2540, MIDUS II age range: 30-84, Mean=55.53, SD=11.21). Perceived discrimination was quantified as the sum of 9 items capturing experiences of daily discrimination. Perceived inequality was measured in three settings: work, family (relationship with children), and home. Both mastery and perceived constraints were used as indicators of control beliefs. The results revealed a significant decline in control between the two waves. Controlling for age, gender, education, self-rated physical health, and baseline control beliefs, higher daily discrimination and perceived inequality were significantly associated with lower control beliefs, 8 to 10 years later. There were not significant interactions with age and the patterns of results persisted when other relevant factors, such as cognitive performance and physical activity, were considered. The findings suggest that interventional programs should target (sources of) perceived discrimination and inequality and that changes in control beliefs could be considered a potential mechanism of the association between social stress and trajectories of healthy aging.

